# Is it possible to model the impact of calorie-reduction interventions on childhood obesity at a population level and across the range of deprivation: Evidence from the Avon Longitudinal Study of Parents and Children (ALSPAC)

**DOI:** 10.1371/journal.pone.0263043

**Published:** 2022-01-31

**Authors:** Simon J. Russell, Steven Hope, Helen Croker, Jessica Packer, Russell M. Viner

**Affiliations:** Obesity Policy Research Unit, Population, Policy and Practice, Great Ormond Street Institute of Child Health, University College London, London, United Kingdom; Erasmus MC, NETHERLANDS

## Abstract

**Background:**

Simulated interventions using observational data have the potential to inform policy and public health interventions where randomised controlled trials are not feasible. National childhood obesity policy is one such area. Overweight and obesity are primarily caused by energy-rich and low-nutrient diets that contribute to a positive net energy imbalance. Using data from the Avon Longitudinal Study of Parents and Children (ALSPAC), we investigated whether causal modelling techniques could be applied to simulate the potential impact of policy-relevant calorie-reduction interventions on population prevalence and inequalities in obesity in childhood.

**Methods:**

Predicted probabilities of obesity at age 11 (UK90 cut offs) were estimated from logistic marginal structural models (MSM) accounting for observed calorie consumption at age 7 and confounding, overall and by maternal occupational social class. A series of population intervention scenarios were modelled to simulate daily calorie-reduction interventions that differed in effectiveness, targeting mechanism and programme uptake level.

**Results:**

The estimated effect of maternal social class on obesity after accounting for confounding and observed calorie intake was provided by the controlled direct effect (CDE), in which, 18.3% of children were living with obesity at age 11 years,. A universal simulation to lower median intake to the estimated average requirement (EAR) (a 6.1% reduction in daily calories) with 75% uptake reduced overall obesity prevalence by 0.6%; there was little impact on inequalities. A targeted intervention to limit consumption to the EAR for children with above average intake reduced population obesity prevalence at 11 years by 1.5% but inequalities remained broadly unchanged. A targeted intervention for children of low-income families reduced prevalence by 0.7% and was found to slightly reduce inequalities.

**Conclusions:**

MSMs allow estimation of effects of simulated calorie-reduction interventions on childhood obesity prevalence and inequalities, although estimates are limited by the accuracy of reported calorie intake. Further work is needed to understand causal pathways and opportunities for intervention. Nevertheless, simulated intervention techniques have promise for informing national policy where experimental data are not available.

## Introduction

Evaluation of large-scale policy interventions is desirable, but challenging. A randomized controlled trial, while considered to be the gold-standard, is seldom feasible, ethical, or sufficiently timely for the investigation of complex policy actions at a population level. However, recent advances in causal inference methods applied to observational datasets provide opportunities to address policy concerns by comparing the likely impact of alternative interventions before they are implemented at scale. Obesity has been the focus of considerable policy attention, as a consequence of rising rates of childhood obesity in the UK and elsewhere. In the UK since 2006/07, National Child Measurement Programme (NCMP) data show obesity rates have increased for children aged 4/5 years and 10/11 years [1]. Obesity tracks from childhood to adolescence and adulthood [2], with associated morbidities including type 2 diabetes and cardiovascular disease [3]. Socioeconomic disadvantage, whether measured by education, occupation or income [4], is strongly linked to increased risks of obesity in childhood [5], with inequalities widening in recent years [1]. Inequalities in obesity are driven by a range of complex determinates: across the socioeconomic gradient there are differences in living conditions, the built environment, in education and employment opportunities, and in access to healthcare services, all of which affect health behaviours, including dietary choices and physical activity [6,7]. The accumulation of excess body fat is caused by energy-rich and low-nutrient diets that result in an energy imbalance [8]; supporting people to reduce calorie intake is one means to reduce BMI.

Reducing population prevalence and inequalities in childhood obesity is high on the public health agenda and is a key priority for policy makers. In the UK, the Childhood Obesity Strategy (2016–2019) acknowledges the many drivers of the problem but also that the ultimate cause of childhood obesity is overconsumption of calories [9]. The strategy comprises a programme of policy goals, which seek to address energy balance by reducing dietary intake and improving diet quality [10]. The policy programme encompasses individuals and families, healthcare systems, and societies. At an individual level, there has been commitment to actions that help people make healthier choices for their families. In terms of communities, there is a particular focus on the school environment, on providing support with the cost of healthy food, and the positive use of available technologies. In terms of healthcare systems, there is a key aim of improving the quality of weight management services in order to provide appropriate treatments and enable people to maintain a healthy weight. Finally, there is a strong focus on structural action, including fiscal measures, regulation of advertising, and challenging industry to improve the nutritional composition and healthiness of food products.

It is widely accepted by policy experts that reducing prevalence and tackling inequalities in obesity requires integrated policy action across individual, community and societal levels [11]. It is also accepted that the complex aetiology of the problem requires interventions that target the structural and socioeconomic determinants of obesity [11]. There is some evidence relating to the effectiveness of preventative and behaviour changing interventions for children of all ages. In terms of preventative interventions, there is weak evidence that dietary interventions alone are effective for any age group and better evidence that dietary and physical activity interventions may be effective in preventing childhood obesity [12]. In terms of individual behavioural interventions, trial evidence from high income countries showed that multi-component behaviour change interventions may achieve reductions in body weight in children of all ages but that the effective components of these interventions remains unclear [13]. The scale up of trial evidence to populations is also problematic given that small scale interventions require effectiveness trials in real world settings and may not function in the same way at a population level [14]. In terms of individual and community interventions that focus on tackling inequalities in childhood obesity, evidence is mixed and UK evidence is low quality [7]. There is also very little evidence as to the effectiveness of policy-based obesity interventions at a population level or relating to societal or multilevel interventions in the UK [15].

There is a need to produce evidence-based policy, despite the challenges of developing and assessing interventions at a population level. Using methods applied elsewhere to the simulation of population interventions [16] this work sought to investigate whether causal modelling techniques could be applied to model the potential population impact of policy action or hypothetical interventions on prevalence and inequalities in obesity. Using longitudinal cohort data, scenarios were modelled to represent reductions in energy intake at age 7y, with estimated impacts on population level obesity at age 11y. Within a social determinants framework, we conceptualised the exposure as an upstream marker of inequalities (maternal social class) which would be associated with obesity through downstream risk factors (mediators, such as energy intake), where the mediator would be the easiest point of policy action. This framing of policymaking as a mediation analysis allowed us to investigate the extent to which downstream policy targets might ameliorate the effects of disadvantage on child obesity.

Given the inequalities in obesity, maternal social class was used as the exposure variable in the model; impacts were reported by level of disadvantage, with relative and absolute inequalities in obesity also estimated. Interventions differed in effectiveness, programme uptake level, were targeted based on income or reported calorie intake levels, or were indicated based on prior weight status.

## Methods

### Data sources

ALSPAC is a geographically defined longitudinal birth cohort in the old administrative county of Avon in Southwest England. Pregnant women resident in Avon, UK with expected dates of delivery 1st April 1991 to 31st December 1992 were invited to take part in the study. The initial number of pregnancies enrolled was 14541. Of these initial pregnancies, there was a total of 14676 foetuses, resulting in 14062 live births and 13988 children who were alive at 1 year of age [17,18].

When the oldest children were approximately 7 years of age, an attempt was made to bolster the initial sample with eligible cases who had failed to join the study originally. As a result, when considering variables collected from the age of 7 onwards (and potentially abstracted from obstetric notes) there are data available for more than the 14541 pregnancies mentioned above. The number of new pregnancies not in the initial sample (known as Phase I enrolment) that are currently represented on the built files and reflecting enrolment status at the age of 24 is 913 (456, 262 and 195 recruited during Phases II, III and IV respectively), resulting in an additional 913 children being enrolled. The phases of enrolment are described in more detail in the cohort profile paper and its update [17]. The total sample size for analyses using any data collected after the age of seven is therefore 15454 pregnancies, resulting in 15589 foetuses. Of these 14901 were alive at 1 year of age.

ALSPAC is a dataset which has frequent follow ups, and includes 34 child-completed questionnaires and 25 mother- or caregiver-completed questionnaires [17]. The ALSPAC study website contains details of all data available through a fully searchable data dictionary and variable search tool [19]. Ethical approval for the study was obtained from the ALSPAC Ethics and Law Committee and the Local Research Ethics Committees. Informed consent for the use of data collected via questionnaires and clinics was obtained from participants following the recommendations of the ALSPAC Ethics and Law Committee at the time.

### Analytic sample

Observations without a unique pregnancy identifier, twins, any child not alive at year one, and observations where consent was withdrawn were deleted, leaving a sample of 14304 children. There were complete data on total daily calories for 7081 children at age 7 (49.5% of the working sample); 3326 had missing data for confounding or outcome variables, resulting in a complete case sample of 3755 children.

The analytic sample included all children with data on the exposure variable (maternal social class; n = 10680). Multiple imputation by chained equations was used to deal with missing data and attrition; fifty datasets were generated, assuming that data were missing at random (see S1 Appendix) [20].

### Measures

Exposure (maternal social class)

Maternal occupational social class recorded at baseline (32 weeks gestation) was used as a measure of household deprivation. It comprised six categories, which were collapsed into three groups for these analyses: high social class (professional, managerial and technical), middle social class (skilled non-manual), and low social class (skilled manual, part-skilled and unskilled).

Outcome (obesity)

Objectively measured height and weight was recorded at age 11 years. Height was measured to the nearest 0.1cm using a Harpenden stadiometer and weight was measured to the nearest 0.1 kg using a Tanita scale [21]. Z-scores were calculated for BMI at age 11 years using the UK90 reference data [22] and cut offs for overweight (>85th percentile) and obesity (>95th percentile) for epidemiological application [23] were applied.

Mediator (total daily calorie consumption)

Total daily calorie intake at age 7 years was calculated from mother-reported three-day diet diaries and subsequent clinic assessments. Parents were sent structured diaries to record all food and drink consumed by the child over two week days and one weekend day. Completed diaries were brought to the clinic and interviews were carried out with parents if clarification was required or if there were anomalies [24,25]. Portion size and nutrient content were calculated for all items in the diary, from which average total daily calorie intake for each child was estimated [26,27]. The 5th edition of McCance and Widdowson’s food tables [28] and supplementary volumes were used for the nutrient analysis of diet diaries. Observations which were beyond the estimated range of normal measurement error, deduced by 95% confidence intervals, were excluded [29,30].

Estimated average requirements (EAR) was used as an estimate of food energy needs for children aged 7 years [31]. The EAR is derived by multiplying basal metabolic rate (BMR) and physical activity level (PAL), accounting for growth and development [31]. A measure of effective and healthy long-term weight loss was provided by applying recommendations for adults [32].

#### Confounding

**Fig 1:**
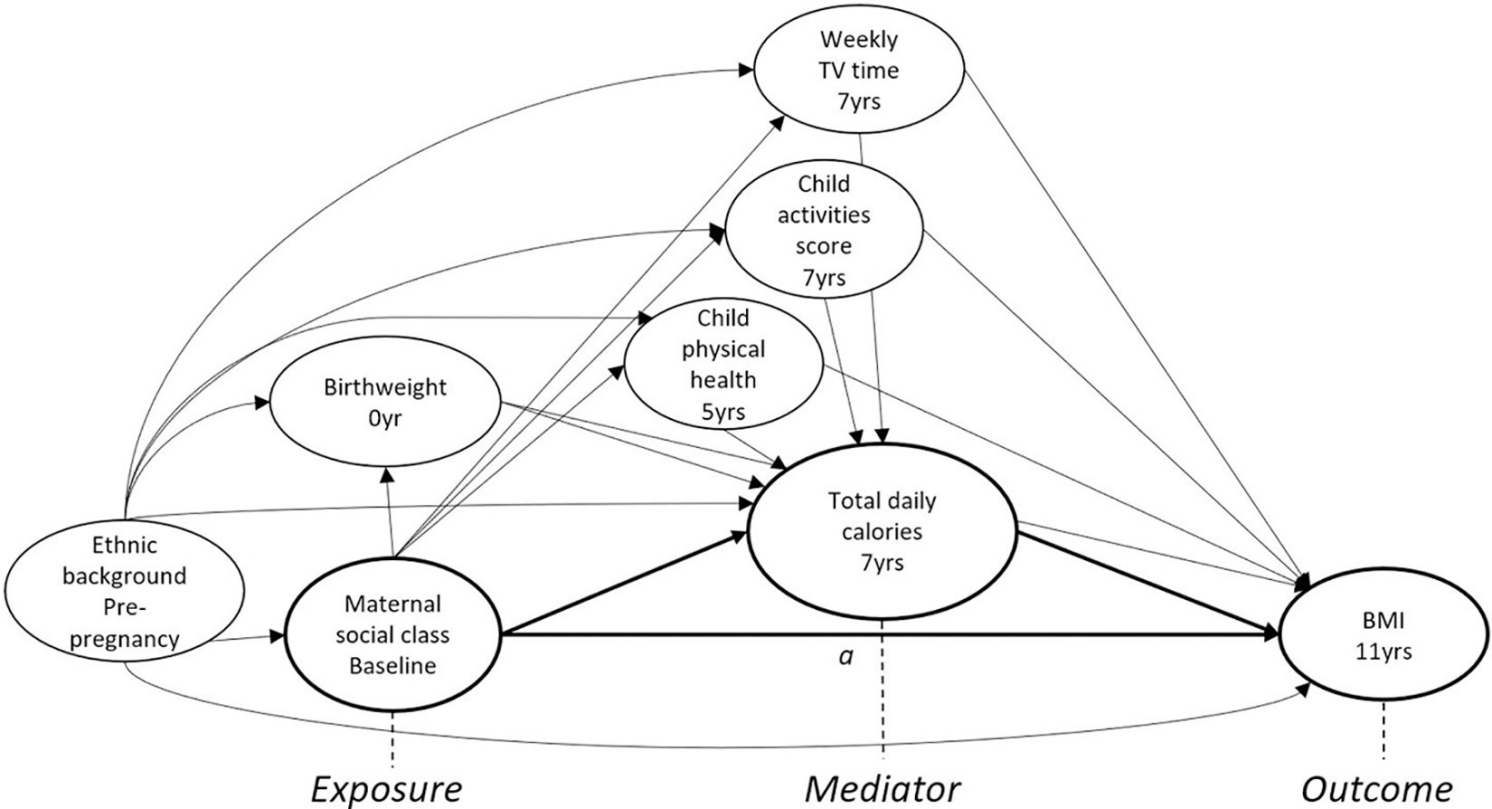
Directed acyclic graph showing theoretical associations between exposure (maternal social class at 0 years), mediator (total daily calories at 7 years) and outcome (BMI at 11 years). ^a^ The controlled direct effect was provided after adjusting total daily calories (accounting for baseline and intermediate confounding) and estimating the effect of maternal social class on obesity. Intervention scenarios were modelled by simulating reductions to calorie intake (varying in effectiveness, targeting and uptake) with impacts on population prevalence and inequalities in obesity observed.

The causal diagram showing the hypothesised relationship between socioeconomic circumstances, calorie intake and childhood obesity, accounting for confounding (Fig 1).

Baseline confounding

Child ethnicity, categorised as ‘white’ and ‘non-white’.

Intermediate confounding

Birthweight, recorded in grams from obstetric data, standardised and categorised as low (<1SD below the mean), mid (-1 to 1), or high (less than 1SD above the mean); physical health of the child at 5 years, re-categorised as generally healthy and sometimes/almost always unwell; child activity score (a sum score provided by ALSPAC of various activities, classes, clubs or groups attended by the child) at 7 years re-categorised from 0–60 into three groups based on the distribution: low (1–10), mid (11–20) and high (21+); and child’s weekly TV time at 7 years, combining typical weekday and weekend day TV viewing into three categories: low (less than or equal to 14 hours per week), mid (between 14 and 26 hours) and high (greater than 26 hours per week). All confounders except birthweight were maternal-report.

#### Data analysis

Descriptive analyses using linear, logistic and multinomial regression were conducted to assess relationships between maternal social class (exposure at baseline), total daily calories (mediator at age 7 years), BMI (outcome at age 11 years), and confounders. Total daily calories were not normally distributed; therefore, median daily calories were reported and used to inform simulations (Table 2). Prevalence of obesity at age 11 years by maternal social class was reported with relative and absolute inequalities (Table 3). Stata SE 15.1 was used to perform all analyses [33].

Logistic regression was used to model the association between maternal social class and BMI at 11 years within a marginal structural modelling (MSM) framework [34]. Inverse probability weights (IPWs) were constructed between the exposure with baseline confounding (weight 1) [34]. This weight represented the change in probability of social class after adjustment for baseline confounders. Weights were stabilised and trimmed (between 1% and 99%) to deal with the potential influence of outliers (see S2 Appendix) [16]. Predicted probabilities obtained from the regression models were used to estimate the prevalence of obesity overall and by maternal occupational social class. This provided the total direct effect (TDE) of maternal social class on obesity [16].

Relative and absolute inequalities (differences in obesity prevalence by social class) were estimated by repeating regression models using maternal occupational social class as a continuous term. Relative inequalities (risk ratios) were given by the ratio of fitted probabilities of overweight and obesity between the highest and lowest maternal occupational social groups, while absolute inequalities (risk differences) were given by the difference between the fitted probabilities between the highest and lowest maternal occupational social groups.

Total daily calories was then included in the model as a continuous variable with IPWs for baseline and intermediate confounding (weight 2). This weight represented the change in probability of calorie intake given social class after adjustment for baseline and intermediate confounders. This provided the controlled direct effect (CDE) from the adjusted model i.e. the estimated effect of maternal social class on obesity when total daily calories was fixed at observed levels [16,35]. The final weight for the CDE was given by multiplying weights 1 and 2. The CDE was the model against which simulated scenarios were compared.

To simulate intervention scenarios, the mediator was adjusted to reflect reductions in calorie intake. Predicted probabilities of obesity prevalence and inequalities were re-estimated for each intervention scenario and compared to the original CDE model.

Effectiveness for intervention scenarios was modelled by simulating various reductions to calorie intake. For each reduction in calories, a normal distribution was generated around the adjusted level in order that likely variability was reflected, meaning reduction of intake varied between individual children receiving the intervention.

Interventions were either universal (for all children; scenario 1), targeted (based on elevated family or individual risk of future obesity; scenarios 2 and 4) or indicated on past weight status (scenario 3). Family based targeting was income-based, where children of low income families (less than 60% the UK median [36,37]) received an intervention (scenario 2) or individual-based, where children with reportedly high consumption received an intervention (scenario 4).

An indicated intervention (scenario 3) was simulated for children living with obesity at a prior age. Obesity at age 7 was defined using z-scores BMI using the UK90 reference data [22] and cut-offs for epidemiological application [23].

In each scenario, only eligible children received an intervention. In the universal scenario, all children were eligible for an intervention but in scenarios 2–4 eligibility was determined according to targeted and indicated criteria.

In terms of uptake of eligible children, for simulations 1 and 2, 75% of children were randomly assigned to the intervention group given that compliance with an intervention would be less than 100%; however, there was no evidence of relevant population interventions to guide a realistic uptake level. When targeting on individual risk (scenario 4) and with the indicated intervention (scenario 3), all children with high consumption or living with obesity at an earlier age were eligible for an intervention.

### Simulations (Table 1)

Each scenario represents a potential population policy action or intervention and follows a structure with a level of effectiveness (extent of calorie reduction), targeting or indicating based on income or risk of future obesity, and a level of uptake of eligible children.

**Table 1:** Simulated intervention scenarios.

Scenarios	Calorie reduction	Target	Uptake
1. Universal intervention to meet estimated average requirements (ear)	-6.1% (-4.8% for boys, -7.5% for girls)	All children	75%
2. Targeted intensive intervention for children of low income families	-21.3%	Children from low income families (33.7%)	75%
3. Indicated intensive intervention for children with prior obesity	-21.3%	Children living with overweight or obesity at age 7 years (9.3%)	100%
4. Targeted intervention for children consuming excess total daily calories	Variable	Boys consuming >1649 kcal per day (55.7%) and girls consuming >1530 kcal per day (70.7%) (62.9% overall)	100%

Scenario 1 modelled the impact of a universal intervention that reduced population intake of calories for children aged 7 years; uptake of the intervention was set at 75%. The population distribution of intake was shifted down by reducing median calorie intake to the EAR. Median intake in calories (taken from food diaries) was 1732.4 for boys and 1654.1 for girls, while the EAR is estimated to be 1649 for boys and 1530 for girls. Therefore, to bring median intake in line with EAR, boys would need to consume 83.4 less calories (4.8%) and girls 124.1 (7.5%), which equated to a 6.1% overall decrease overall. For boys, this daily decrease is approximately equivalent to a fun size Mars bar and for girls a fun size Mars bar plus a Maryland cookie. Sex-specific reductions were applied with random variation.

Scenario 2 modelled a more intensive intervention and was targeted to children of low-income families; uptake among eligible children was set at 75%. The intensive intervention was designed to represent a healthy weight loss intervention and was informed by EAR values for adults (sex and age groups combined) [31] and the recommendation for adults to eat 500 calories fewer per day to achieve healthy, long-term weight loss [32]. Based on this, the effectiveness of the hypothetical intervention, equated to a 21.3% decrease in daily intake. Given the relationship between disadvantage and obesity [5], children from low-income families were targeted as they are at heightened risk.

Scenario 3 modelled the same intensive reductions in intake based on recommendations for effective, healthy and long-term weight loss in adults (as in scenario 2, this equated to a -21.3% decrease in daily intake). This intervention was for children living with obesity at a prior time, given that obesity in children tracks through adolescence and into adulthood [2]. Eligible children were indicated using weight status; uptake (all children living with obesity at age 7 years) was 9.3%. Scenarios 2 and 3 represented a substantial reduction in daily calories and were only applied to children living with or at elevated risk of obesity.

Scenario 4 modelled the impact of a targeted intervention that reduced intake among children reported to be consuming the most. This intervention effectively truncated the population distribution of intake by targeting children with intake exceeding EAR. For these children, intake was then fixed at the level of the EAR in order that every child’s intake was equal to or less than the EAR (1649 and 1530 daily calories for boys and girls respectively).

For all simulations, as guided by the lower nutrient intake bound [38], a lower bound was set at 2SD below the mean intake as reported from food diaries in the ALSPAC analytic sample. This lower bound prevented simulations from reducing calorie intakes for children with low reported consumption. Variables used in targeted and indicated scenarios were recorded at the same time as the mediator and were independent to the model.

#### Sensitivity analyses

Four sensitivity analyses were carried out. In the first, the models were estimated in a complete case sample (n = 3755). In the second, analyses were repeated using maternal education as an alternative measure of socioeconomic circumstances. In the third, simulations were repeated with higher and lower levels of uptake (100% and 30%) to represent complete and lower compliance with a policy change or hypothetical intervention; there was no clear evidence to inform compliance with interventions so we sought to describe a pattern of results across varying uptake levels. Fourth, simulations were repeated with an outcome of combined overweight and obese categories.

## Results

### Descriptives

Of mothers in the analytic sample, 25.7% were from the lowest social class (comprising ‘skilled manual’, ‘part-skilled’ and ‘unskilled’ occupations; Table 2). At age 7, median daily calorie consumption was 1732.4 for boys and 1654.1 for girls, which was 83.4 and 124.1 calories higher than the EAR respectively. The percentage of children with daily intake equal to or less than the EAR was 44.3% and 29.3% for boys and girls respectively. Obesity prevalence at 11 years was 18.3%.

**Table 2:** Descriptive statistics of ALSPAC across analytical samples.

		Whole sample n = 14,304	Complete Case n = 3,652	Imputed sample (m = 50) n = 10,680
Sex	Male	(7330) 51.2%	(1843) 50.5%	51.9%
	Female	(6974) 48.8%	(1809) 49.5%	48.1%
**Exposure**				
Mother Social Class	Low	(2749) 25.7%	(671) 18.4%	25.7%
	Mid	(4561) 42.7%	(1531) 41.9%	42.7%
	High	(3370) 31.6%	(1450) 39.7%	31.6%
	Missing	(3,624)	-	-
**Baseline confounding (0 years)**				
Ethnicity	White	(11173) 95.0%	(3558) 97.4%	95.7%
	Non-white	(591) 5.0%	(94) 2.6%	4.3%
	Missing	(2540)	-	-
**Mediator**				
Total daily calories	Median kcal (SE)	1702.8 (3.8)	1700.3 (6.3)	1694.3 (5.3)
	Missing	(7223)	-	-
**Intermediate confounding**				
Birthweight	Low	(1850) 13.3%	(414) 11.3%	13.2%
	Mid	(10176) 73.2%	(2668) 73.1%	72.3%
	High	(1869) 13.5%	(570) 15.6%	14.5%
	Missing	(409)	-	-
Child physical health	Very healthy/healthy	(8614) 97.0%	(3565) 97.6%	97.1%
	Sometimes/always unwell	(265) 3.0%	(87) 2.4%	2.9%
	Missing	(5425)	-	-
Child activities score	Low	(624) 7.3%	(210) 5.8%	7.2%
	Mid	(5638) 66.3%	(2364) 64.7%	66.4%
	High	(2238) 26.3%	(1078) 29.5%	26.4%
	Missing	(5,804)	-	-
TV time	Low (< = 14hrs)	(736) 9.1%	(347) 9.5%	7.6%
	Mid (>14hrs and < = 26hrs)	(6382) 78.8%	(2982) 81.7%	80.9%
	High (>26 hours)	(977) 12.1%	(323) 8.8%	11.6%
	Missing	(6209)	-	-
**Outcome**				
BMI status (11 years)	Not obese	(4531) 65.5%	(2444) 66.9%	65.6%
	Overweight (85^th^-95^th^)	(1056) 15.3%	(587) 16.1%	16.1%
	Obese (>95^th^ centile)	(1327) 19.2%	(621) 17.0%	18.3%
	Missing	(7390)	-	-
**Informing/targeting variables for interventions**				
BMI status (7 years)	Not overweight/obese	(6316) 79.1%	(2951) 80.8%	79.0%
	Overweight (85^th^-95^th^)	(856) 10.7%	(391) 10.7%	11.7%
	Obese (>95^th^)	(809) 10.1%	(310) 8.5%	9.3%
	Missing	(6323)	-	-
Income	1 <£100pw	(301) 4.1%	(61) 1.9%	4.0%
	2 £100-£199pw	(834) 11.3%	(256) 7.8%	11.6%
	3 £200-£299pw	(1350) 18.3%	(558) 17.1%	19.1%
	4 £300-£399pw	(1649) 22.4%	(791) 24.2%	22.9%
	5 >£400pw	(3235) 43.9%	(1605) 49.1%	42.4%
	missing	(6935)	-	-

Maternal social class at 32 weeks gestation predicted obesity at age 11 years. Compared to children from the highest social class, those from the lowest social class were 1.4 times more likely to be living with obesity. Total daily calories reported at age 7 years was positively associated with increasing BMI z-scores and significantly predicted obesity at age 11 years (p<0.001). There was a weak relationship between maternal occupational class and dietary intake where the highest daily calorie consumption was reported among the highest social class group (1712.1 kcals, SE = 8.4) compared to the middle and lower social class groups (1686.1 kcals, SE = 12.9 and 1685.5 kcals, SE = 10.6 respectively; S1 and S2 Tables).

### Simulated interventions

Table 3 summarises the estimated prevalences by social class and absolute and relative inequalities, for each scenario with comparison to the CDE. We tested for a multiplicative interaction between exposure and mediator and found it was not significant; therefore, we did not include an interaction term in the model or separately estimate the CDE for each level of exposure. However, we acknowledge that, in situations where the exposure and mediator are both associated with the outcome, an interaction will be observed on at least one scale. In this case, indicating that an additive interaction will be present, which has not been accounted for in these models.

**Table 3:** Prevalence of obesity at age 11 years by maternal social class with risk ratios and differences for relative and absolute inequalities, for intervention scenarios 1–6.

Scenario	Consuming < = EAR (boys/girls)	Prevalence of obesity at 11 years (> = 95^th^ centile)				Inequalities in obesity^a^	
		Overall (change vs CDE)	Maternal occupational social class			Risk ratio^b^ (CIs)	Risk difference^b^ (CIs)
			Low (change vs CDE)	Mid (change vs CDE)	High (change vs CDE)		
Controlled Direct Effect^c^							
	44.3%/29.3%	18.3%	20.3%	18.2%	16.7%	1.21 (1.06–1.36)	3.52 (1.15–5.88)
Simulation 1: Universal intervention to meet kcal per day recommendation (-6.1% overall), 75%							
	54.6%/39.3%	17.7% (-3.5%)	19.6% (-3.5%)	17.6% (-3.5%)	16.2% (-3.5%)	1.21 (1.06–1.37)	3.41 (1.11–5.71)
Simulation 2: Targeted intensive intervention (-21.3%) for children from low income families (35.0%), 75% uptake overall							
	55.5%/41.8%	17.6% (-3.7%)	19.3% (-4.8%)	17.6% (-3.8%)	16.3% (-2.6%)	1.18 (1.03–1.33)	2.98 (0.69–5.27)
Simulation 3: Indicated intensive intervention (-21.3%) for children with obesity, 9.3% uptake							
	48.5%/33.7%	18.0% (-1.4%)	19.9% (-1.6%)	18.0% (-1.4%)	16.5% (-1.3%)	1.21 (1.05–1.36)	3.41 (1.08–5.75)
Simulation 4: Targeted calorie-reduction simulation for children consuming excess total daily calories to limit intake to EAR, 62.6% uptake overall							
	100%/100%	16.8% (-8.3%)	18.7% (-8.0%)	16.8% (-8.0%)	15.3% (-8.8%)	1.22 (1.06–1.38)	3.38 (1.17–5.60)

^a^ Relative and absolute inequalities were estimated using a continuous linear term for maternal social class.

^b^ Risk ratios and differences are likelihoods calculated with reference to non-obese group (<95^th^ centile of zBMI at age 11 years).

^c^ The effect of maternal social class on obesity prevalence at age 11 years, adjusted for baseline and intermediate confounding with mediation of total daily calories held at observed level.

The CDE estimated population prevalence of obesity for children aged 11 years after adjustment for reported calorie intake at 7 years; inequalities in obesity were observed. There was almost no difference in prevalences between the TDE (effect of household disadvantage on obesity with confounding) and the CDE (including calorie intake in the model), showing that reported intake had little mediating effect on the relationship between maternal occupational class and obesity at age 11 years. The relationship was unmediated by calorie consumption owing to the inverse relationship between intake and disadvantage, where the highest reported intake was observed in the highest social group.

Scenario 1 modelled the impact of a universal calorie reduction of 6.1% with 75% uptake. Compared to the CDE there was a reduction in overall obesity prevalence from 18.3% to 17.7%; i.e. a 3.5% reduction in obesity relative to the CDE. There was little change to relative inequalities, while absolute inequalities were marginally reduced following this intervention. The percentage of children with daily intake equal to or less than the EAR was increased following this simulation to 54.6% and 39.3% for boys and girls respectively.

Scenario 2 modelled a 21.3% reduction in calories, targeted to children of low-income families, with 75% uptake of eligible children. Overall obesity prevalence was reduced from 18.3% to 18.0%; i.e. a 3.7% change compared to the CDE. Relative and absolute inequalities were slightly reduced.

Scenario 3 modelled a 21.3% reduction in calories for children with obese at age 7. Compared to the CDE, estimated prevalence of obesity was slightly reduced from 18.3% to 18.0%; i.e. a 1.4% change compared to the CDE. There was little change in relative inequalities and a marginal narrowing of absolute inequalities.

Scenario 4 modelled a variable reduction in calories, targeted intervention to children with daily intake exceeding the EAR. Limiting maximum intake of all children to the EAR and produced the greatest overall effect, reducing obesity prevalence at 11 years from 18.3% to 16.8%; i.e. an 8.3% change compared to the CDE; there was little change in inequalities. In this scenario, all children had daily intake equal to or less than the EAR.

### Sensitivity analysis

Results from models using maternal education as the exposure were very similar to those reported. Analysis repeating scenario 1 (a universal calorie reduction of 6.1%) but with 100% uptake, demonstrated a stronger pattern of results or lower (30%) uptake, demonstrated a weaker pattern of results. The results were also similar when combined overweight and obesity outcome was used (for sensitivity analyses, S3–S5 Tables).

## Discussion

### Summary

We have shown that it is feasible to use MSM to estimate the effects of interventions to reduce calorie intake at age 7 on BMI at age 11 years, within a social determinants framework where the exposure is occupational social class in early childhood.

Obesity was 18.3% and predicted by disadvantage in children aged 11 years. Children aged 7 were consuming excess calories compared to the estimated average requirement (EAR), according to food diary report. There was an unexpected social patterning of intake across social groups, where the highest intake was reported among the highest social group. Models simulating the effects of reducing calorie consumption at age 7 had some effect on obesity prevalence and reductions were proportional to the effectiveness of the hypothetical intervention. Inequalities in the outcome remained broadly unchanged following interventions; however, decreases in prevalence were greatest in the lower social groups, meaning calorie reduction measures may be impactful for disadvantaged children. The impact of hypothetical interventions was successfully modelled at a population level; however, the reliability of the model and the effect sizes is subject to a number of caveats, including the accuracy of maternal-report of intake from food diaries and the social patterning of intake across social class groups. Further research is needed to better describe causal pathways, exploring the potential impact of timing and duration of an intervention, and the comparative effects on inequalities in obesity of interventions on multiple downstream factors, by including, for example, physical activity in addition to calorie intake.

### Research context

Policy makers in the UK [10] and elsewhere have identified reducing dietary intake and improving dietary quality as key areas for policy action in tackling childhood obesity. However, there are few intervention programmes that operate at a population level. The meaningful extrapolation of small-scale trial evidence to populations is also challenging, given that the effective components of an intervention may be unclear and the contexts in which they are effective may vary [39]. Given the absence of population data and the challenges of extrapolating trial data, we adopted a simulation approach to estimate the potential impacts of hypothetical interventions that result in calorie-reductions on population prevalence and inequalities in childhood obesity. The nature or characteristics of these interventions are not described and could include individual behavioural interventions, such as health promotion initiatives, structural policy actions, such as taxation on unhealthy food and drinks, or be the by-product of broader policy levers.

Universal, targeted and indicated calorie-reduction interventions were found to reduce population prevalence of obesity in children but effect sizes were modest. Given that excess energy intake is the main driver of obesity, we might expect greater impacts on obesity following reductions in dietary intake. That observed changes were modest may be symptomatic of the relationship between obesity and disadvantage, which is likely to increase the risk of weight gain in multifactorial ways. The pathways between disadvantage and obesity may be direct or indirect and may be affected by a range of individual, lifestyle, sociocultural and environmental factors.

Despite the positive association between increasing disadvantage and BMI, the interventions modelled showed that reducing obesity prevalence had little to no effect on inequalities. Since higher energy intake predicts overweight and obesity, we expected disadvantage to predict greater calorie consumption but this trend was not observed. Universal calorie-reduction interventions reduced prevalence but not inequalities in obesity, likely due to the social patterning of reported intake. Despite this, given the higher prevalence of obesity and that prevalence decreases following interventions were greater among lower social groups, targeted interventions for disadvantaged children are likely to narrow inequalities in obesity.

### Strengths and limitations

There is a lack of evidence of the impact of population interventions to address childhood obesity and this is the first study to our knowledge that has simulated calorie reduction interventions at a population level. A number of hypothetical scenarios were modelled that focussed on estimated energy requirements for children, general guidance for healthy weight-loss [32] and policy targets. This study used a large UK regional cohort of children for which the exposure, mediator and outcome variables were recorded temporally. The ALSPAC cohort includes objectively recorded BMI data, reliable dietary intake and a number of potential confounding factors.

There were some limitations of the data. The regional sample was large but not nationally representative of the wider UK population, notably, ALSPAC mothers were more affluent and better educated than the UK average. There was also under-representation of the most deprived groups in the sample population owing to systematic differences between participating mothers and those who did not enrol [18]. Under-representation of the most deprived groups may also have occurred within the sample, given that participants of greater disadvantage were disproportionately lost to attrition [18]. These data may under estimate caloric intake and obesity prevalence for children of lower social class given that rates of drop out are higher among such groups.

The cohort was based on children born in early 90s, and BMI data were generated in 2002. Rates are likely to be lower in the analytic sample compared to current populations, given the trend of increasing obesity since 2002 [1]; it is also likely based on this trend that contemporary UK children consume more calories compared to those in this cohort. Estimate average requirements were also based on relatively recent data of body weights (2011) [31], meaning the average requirement in 2002 is likely to have been a lower estimate. Therefore, the extent that children were overeating based on food diaries was probably underestimated in these analyses with implications for modelled interventions and reductions in overall prevalence.

Estimated intake was based on food diaries, which have been shown to be valid [40] measures of dietary intake and have shown good agreement with total energy expenditure [41]; however, concerns remain about potential report bias [25]. Three-day diet diaries are a snapshot of behaviour and may not represent normal routines or relate to BMI; the act of observing and recording intake may also modify mother or child dietary behaviour. Given well-established inequalities in obesity, we expected to observe a higher daily intake of calories in disadvantaged groups; however, we found that reported intake was similar across the social classes. There is inconsistent or an absence of evidence that calorie consumption in adults is markedly different across social groups but strong evidence that dietary quality is socially patterned and is likely to be a key contributor to inequalities in obesity. Despite some limitations with the estimates, it is likely that the associations between social class, dietary intake and obesity would remain [42,43].

We accounted for a number of potential confounders [44,45] but there are likely to be other factors that we were not able to account. For example, child activities score was used as a proxy measure for activity but was not an objectively recorded measure of physical activity. A limitation of using observational data is that we were limited in the extent that we could infer causation relating to the pathway to obesity, given that there may remain unmeasured confounding factors between the exposure, mediator and outcome relationships [46]. This is particularly relevant to the pathway from social deprivation to BMI, which is complex. However, we were successful in describing a minimally sufficient causal diagram, with adjustment to account for the potential problem of collider bias.

Replicating these models in other cohorts, using more contemporary data and at different ages will increase our understanding of the likely impact of calorie reduction interventions on population prevalence and inequalities of childhood obesity.

### Implications for policy

Causal inference methods provide a means to simulate the implementation and comparison of any number of competing population policy actions, before they are trialled, or rolled out nationally. The adoption of a mediation approach within a social determinants framework allows for the estimation of the impact on prevalence and inequalities in the outcome of realistic targeting of interventions on downstream risk factors, which mediate the effects of poverty on child outcomes.

The simulations presented here indicate that policy actions or interventions that reduce calorie intake in children would be likely to reduce obesity prevalence. The reductions in obesity prevalence following these simulations were small; however, such changes are likely to be highly meaningful at a population level. Approaches to reduce the incidence of disease are more effective when the distribution of a risk factor is reduced by a small amount at a population level, compared to targeting large reductions among individuals at the highest risk [47]. This is particularly true when the population attributable risk is high, as is the case with obesity, given that the prevalence and the likelihood of associated morbidity are high.

Universal interventions were not found to reduce relative or absolute inequalities providing further evidence that the pathway from disadvantage to obesity is complex. However, given that reductions in obesity prevalence following interventions were greatest in the lowest social groups, targeting disadvantaged children would be likely to result in a narrowing of inequalities. A focus on inequalities should remain at the heart of policy seeking to address the childhood obesity problem, given the higher prevalence of obesity in lower social groups, the tracking of obesity through to adulthood and health impacts across the life course.

## Conclusions

The mechanisms by which social disadvantage increases the likelihood of childhood obesity are complex. Using mediation models, we were able to address concerns of real-world relevance, providing evidence to inform policy decisions on the potential impact of changes in calorie intake on population prevalence and inequalities in obesity.

## Supporting information

S1 Appendix Further details of multiple imputation. (DOCX)

S2 Appendix Boxplot, mean and range for IPWs, before and after stabilisation (using MI data). (DOCX)

S1 Table Relationship between maternal social class and median total daily calories and obesity (n = 10,680). (DOCX)

S2 Table Relationship between baseline and time-varying confounding and exposure, mediator and outcome variables (n = 10,680). (DOCX)

S3 Table CDE and simulation 1 by highest level of maternal education (n = 10,680). (DOCX)

S4 Table CDE and simulation 1 by 100%, 75% and 30% uptake (n = 10,680). (DOCX)

S5 Table CDE and simulation 1 for obesity and overweight/obesity combined (n = 10,680). (DOCX)

## References

[1] National Child Measurement Programme. National Child Measurement Programme–England, 2018/19: Tables. [last accessed 20/03/2020] https://digital.nhs.uk/data-and-information/publications/statistical/national-child-measurement-programme/2018-19-school-year. 2020.

[2] Simmonds M, Llewellyn A, Owen CG, Woolacott N. Predicting adult obesity from childhood obesity: a systematic review and meta-analysis. Obesity Reviews. 2015; doi: 10.1111/obr.12334 26696565

[3] Abdelaal M, le Roux CW, Docherty NG. Morbidity and mortality associated with obesity. Annals of translational medicine. 2017;5(7):161. doi: 10.21037/atm.2017.03.107 28480197 PMC5401682

[4] Devaux M, Sassi F. Social inequalities in obesity and overweight in 11 OECD countries. European Journal of Public Health. 2011;23(3):464–469. doi: 10.1093/eurpub/ckr058 21646363

[5] El-Sayed AM, Scarborough P, Galea S. Socioeconomic Inequalities in Childhood Obesity in the United Kingdom: A Systematic Review of the Literature. Obesity Facts. 2012;5:671–692. doi: 10.1159/000343611 23108336

[6] Loring B, Robertson A. Obesity and inequities: Guidance for addressing inequities in overweight and obesity. [last accessed 18/03/2020] http://www.euro.who.int/__data/assets/pdf_file/0003/247638/obesity-090514.pdf. ISBN 978 92 890 5048 7. 2014.

[7] Bambra CL, Hillier FC, Cairns J, Kasim A, Moore HJ, Summerbell CD. How effective are interventions at reducing socioeconomic inequalities in obesity among children and adults? Two systematic reviews. Public Health Research. 2015;3.1. doi: 10.3310/phr03010 25654155

[8] Rush EC, Yan MR. Evolution not Revolution: Nutrition and Obesity. Nutrients. 2017;9(5):519. 10.3390/nu9050519 PMC5452249 28531097

[9] Department of Health and Social Care. Childhood obesity: a plan for action. Chapter 2. [last accessed 02/02/2020] https://assets.publishing.service.gov.uk/government/uploads/system/uploads/attachment_data/file/718903/childhood-obesity-a-plan-for-action-chapter-2.pdf. 2016.

[10] Department of Health and Social Care. Advancing our health: prevention in the 2020s. [last accessed 02/02/2020] https://assets.publishing.service.gov.uk/government/uploads/system/uploads/attachment_data/file/819766/advancing-our-health-prevention-in-the-2020s-accessible.pdf. 2019.

[11] Butland B, Jebb S, Kopelman P, McPherson K, Thomas S, Mardell J, et al. Tackling Obesities: Future Choices—Project Report. London: Government Office for Science; 2007.

[12] Brown T, Moore THM, Hooper L, Gao Y, Zayegh A, Ijaz S, et al. Interventions for preventing obesity in children. Cochrane Systematic Review. 2019; doi: 10.1002/14651858.CD001871.pub4 31332776 PMC6646867

[13] Ells LJ, Rees K, Brown T, Mead E, Al-Khudairy L, Azevedo L, et al. Interventions for treating children and adolescents with overweight. International Journal of Obesity. 2018;42:1823–1833. doi: 10.1038/s41366-018-0230-y 30301964

[14] Pfadenhauer LM, Gerhardus A, Mozygemba K, Lysdahl KB, Booth A, Hofmann B, et al. Making sense of complexity in context and implementation: the context and implementation of complex interventions (CICI) framework. Implementation Sci. 2017;12,21; doi: 10.1186/s13012-017-0552-5 28202031 PMC5312531

[15] Hillier-Brown FC, Bambra CL, Cairns JM. A systematic review of the effectiveness of individual, community and societal level interventions at reducing socioeconomic inequalities in obesity amongst children. BMC Public Health. 2014;14:834. doi: 10.1186/1471-2458-14-834 25113624 PMC4137097

[16] Pearce A, Hope S, Griffiths L, Cortina-Borja M, Chittleborough C, Law C. What if all children achieved WHO recommendations on physical activity? Estimating the impact on socioeconomic inequalities in childhood overweight in the UK Millennium Cohort Study. International Journal of Epidemiology. 2018; doi: 10.1093/ije/dyy267 30535024 PMC6380318

[17] Fraser A, Macdonald-Wallis C, Tilling K, Boyd A, Golding J, Smith GD, et al. Cohort Profile: The Avon Longitudinal Study of Parents and Children: ALSPAC mothers cohort. International Journal of Epidemiology. 2013;42:97–110. doi: 10.1093/ije/dys066 22507742 PMC3600619

[18] Boyd A, Golding J, Macleod J, Lawlor DA, Fraser A, Henderson J, et al. Cohort Profile: The ‘Children of the 90s’; the index offspring of The Avon Longitudinal Study of Parents and Children (ALSPAC). International Journal of Epidemiology. 2013;42:111–127. doi: 10.1093/ije/dys064 22507743 PMC3600618

[19] ALSPAC. Explore data and samples. [last accessed 08/10/2019] http://www.bristol.ac.uk/alspac/researchers/our-data/. 2019.

[20] Azur MJ, Stuart EA, Frangakis C, Leaf PJ. Multiple Imputation by Chained Equations: What is it and how does it work? International Journal of Methods in Psychiatric Research. 2012;20(1):40–49. 10.1002/mpr.329 PMC3074241 21499542

[21] Bell JA, Carslake D, O’Keeffe LM, Frysz M, Howe LD, Hamer M, et al. Associations of Body Mass and Fat Indexes With Cardiometabolic Traits. Journal of the American College of Cardiology. 2018; doi: 10.1016/j.jacc.2018.09.066 30545453 PMC6290112

[22] Cole TJ, Freeman JV, Preece MA. Body mass index reference curves for the UK, 1990. Archives of Disease in Childhood. 1995;73:25–29. doi: 10.1136/adc.73.1.25 7639544 PMC1511150

[23] Troiano RP, Flegal KM, Kuczmarski RJ, Campbell SM, Johnson CL. Overweight prevalence and trends for children and adolescents: the National Health and Nutrition Examination Surveys, 1963 to 1991. Archives of Pediatrics and Adolescent Medicine. 1995;149:1085–1091. doi: 10.1001/archpedi.1995.02170230039005 7550810

[24] Bull CJ, Northstone K. Childhood dietary patterns and cardiovascular risk factors in adolescence: results from the Avon Longitudinal Study of Parents and Children (ALSPAC) cohort. Public Health Nutrition. 2016;19(18):3369–3377. doi: 10.1017/S1368980016001592 27339189 PMC5197929

[25] Emmett P. Dietary assessment in the Avon Longitudinal Study of Parents and Children. European Journal of Clinical Nutrition. 2009; 63. Suppl. 1: S38–S44. doi: 10.1038/ejcn.2008.63 19190642

[26] Wrieden WL, Longbottom PJ, Adamson AJ, Ogston SA, Payne A, Haleem MA, et al. Estimation of typical food portion sizes for children of different ages in Great Britain. British Journal of Nutrition. 2008;99:1344–1353. doi: 10.1017/S0007114507868516 18031591

[27] Gregory J, Lowe S. National Diet and Nutrition Survey: young people aged 4–18 years. Vol. 1, Report of the diet and nutrition survey. The Stationery Office: London. 2000.

[28] Holland B, Welch AA, Unwin ID, Buss DH, Paul AA, Southgate DAT. The Composition of Foods. 5th ed. The Royal Society of Cambridge; Cambridge. 1991.

[29] Glynn L, Emmett P, Rogers I. Food and nutrient intakes of a population sample of 7‐year‐old children in the south‐west of England in 1999/2000 –what difference does gender make? Journal of Human Nutrition and Dietetics. 2005;18:7–19. doi: 10.1111/j.1365-277X.2004.00582.x 15647094

[30] Johnson L, Mander AP, Jones LR, Emmett PM, Jebb SA. A prospective analysis of dietary energy density at age 5 and 7 years and fatness at 9 years among UK children. International Journal of Obesity. 2007;32:586–593. doi: 10.1038/sj.ijo.0803746 17912267

[31] SACN. Dietary Reference Values for Energy. Scientific Advisory Committee on Nutrition. London TSO. www.sacn.gov.uk. 2011.

[32] American Medical Association. Food and Nutrition: Healthy Weight Loss. JAMA. 2014;312.9:974. 10.1001/jama.2014.10929 25182116

[33] StataCorp. Stata Statistical Software: Release 15. College Station, TX: StataCorp LLC. 2017.

[34] Robins JM, Hernan MA, Brumback B. Marginal structural models and causal inference in epidemiology. Epidemiology. 2000;11:550–560. doi: 10.1097/00001648-200009000-00011 10955408

[35] VanderWeele TJ. Controlled direct and mediated effects: definition, identification and bounds. Scandinavian Journal of Statistics, Theory and Applications. 2011;38(3):551–563. 10.1111/j.1467-9469.2010.00722.x PMC4193506 25309023

[36] Department for work and pensions. Households Below Average Income: An analysis of the income distribution 1994/95–2013/14. [last accessed 05/12/19] https://assets.publishing.service.gov.uk/government/uploads/system/uploads/attachment_data/file/437246/households-below-average-income-1994-95-to-2013-14.pdf. 2015.

[37] Department for work pensions. How low income is measured in households below average income. [last accessed 05/12/19] https://www.gov.uk/government/publications/how-low-income-is-measured/text-only-how-low-income-is-measured. 2016.

[38] Department of Health. Dietary Reference Values for Food Energy and Nutrients for the United Kingdom–Report of the Panel on Dietary Reference Values of the Committee on Medical Aspects of Food Policy. London HMSO. 1991. 1961974

[39] Bonell C, Fletcher A, Morton M, Lorenc T, Moore L. Realist randomised controlled trials: a new approach to evaluating complex public health interventions. Social Science and Medicine. 2012;75:2299–2306. doi: 10.1016/j.socscimed.2012.08.032 22989491

[40] Gibney M, Margetts B, Kearney J, et al. Public Health Nutrition. Blackwell Publishing; Oxford. 2004.

[41] Lennox A, Bluck L, Page P, Pell D, Cole D, Ziauddeen N, et al. Misreporting in the National Diet and Nutrition Survey Rolling Programme (NDNS RP): Summary of results and their interpretation. Appendix X, National Diet and Nutrition Survey (2008/2009–2011/12). 2012.

[42] Giskes K, Avendano M, Brug J, Kunst AE. A systematic review of studies on socioeconomic inequalities in dietary intakes associated with weight gain and overweight/obesity conducted among European adults. Obesity Reviews. 2010;11(6):413–429. doi: 10.1111/j.1467-789X.2009.00658.x 19889178

[43] Darmon N, Drewnowski A. Does social class predict diet quality? The American Journal of Clinical Nutrition. 2008;87(5):1107–1117. doi: 10.1093/ajcn/87.5.1107 18469226

[44] Katzmarzyk PT, Barreira TV, Broyles ST, Champagne CM, Chaput JP, Fogelholm M, et al. Physical activity, sedentary time, and obesity in an international sample of children. Medicine & Science in Sports & Exercise. 2015;47(10):2062–2069. 25751770 10.1249/MSS.0000000000000649

[45] Reilly JJ, Penpraze V, Hislop J. Objective measurement of physical activity and sedentary behaviour: review with new data. Archives of Disease in Childhood. 2008;93:614–619. doi: 10.1136/adc.2007.133272 18305072

[46] Valente MJ, Pelham WE III, Smyth H, MacKinnon DP. Confounding in Statistical Mediation Analysis: What It Is and How to Address It. Journal of Counseling Psychology. 2017;64(6):659–671. doi: 10.1037/cou0000242 29154577 PMC5726285

[47] Rose G. Strategy of prevention: lessons from cardiovascular disease. The British Medical Journal (Clinical research Ed). 1981;282(6279):1847–1851. doi: 10.1136/bmj.282.6279.1847 6786649 PMC1506445

