# Supplementary material for: Is it possible to model the impact of calorie-reduction interventions on childhood obesity at a population level and across the range of deprivation: Evidence from the Avon Longitudinal Study of Parents and Children (ALSPAC)
Source: PLoS One. 2022 Jan 31;17(1):e0263043. doi: 10.1371/journal.pone.0263043 (PMC8803143; doi:10.1371/journal.pone.0263043)
Supplement: S1 Appendix — (DOCX) [file pone.0263043.s001.docx]

**S1 Appendix:** Further details of multiple imputation

Maternal age, education and social class were registered as regular variables, in addition to household income at age 7 years, which was used to inform disproportionate uptake scenarios, and BMI at age 7 years, which was used for a targeted intervention. Mother-reported marital status, collected at 8 weeks gestation, and mother age at time of delivery, were used as auxiliary variables for the multiple imputation. Both had complete or near complete observations and were significant in predicting missingness for the mediator and confounding variables.

Data were imputed using logistic regression for ethnicity and child’s physical health; ordered logistic regression for household income; multinomial regression for mothers marital status; predictive mean matching (to one of ten neighbouring data points) for childhood activities score; and linear regression for zBMI at 7 and 11 years, birthweight, weekly TV time and total daily calories. Augmented regression was used to overcome the presence of empty cells.

Imputed datasets were broadly consistent with the complete case sample in terms of frequencies and summary statistics.
