# Supplementary material for: Is it possible to model the impact of calorie-reduction interventions on childhood obesity at a population level and across the range of deprivation: Evidence from the Avon Longitudinal Study of Parents and Children (ALSPAC)
Source: PLoS One. 2022 Jan 31;17(1):e0263043. doi: 10.1371/journal.pone.0263043 (PMC8803143; doi:10.1371/journal.pone.0263043)

**S2 Appendix:** Boxplot, mean and range for IPWs, before and after stabilisation (using MI data)

Boxplot of IPW before stabilisation (w) (mean = 1.0 [range 0.2 – 4.9])


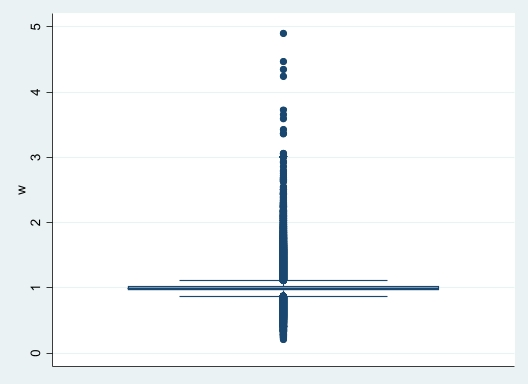


Boxplot of IPW before stabilisation (w99) (mean = 1.0 [range 0.7 – 1.4])


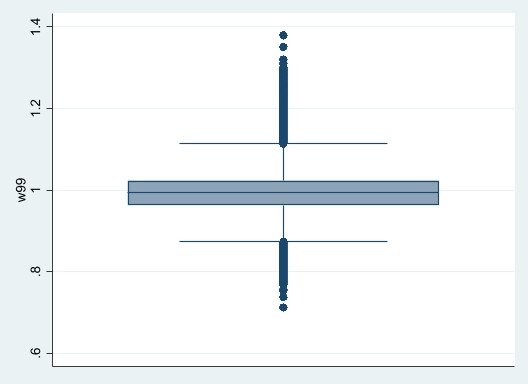

Supplement: S2 Appendix — (DOCX) [file pone.0263043.s002.docx]
