# Supplementary material for: Is it possible to model the impact of calorie-reduction interventions on childhood obesity at a population level and across the range of deprivation: Evidence from the Avon Longitudinal Study of Parents and Children (ALSPAC)
Source: PLoS One. 2022 Jan 31;17(1):e0263043. doi: 10.1371/journal.pone.0263043 (PMC8803143; doi:10.1371/journal.pone.0263043)
Supplement: S1 Table — (DOCX) [file pone.0263043.s003.docx]

**S1 Table.** Relationship between maternal social class and median total daily calories and obesity (n=10,680)

| **Maternal social class** | **Median total daily calories (SE)** | **zBMI 11 years**  **% obese** |
| --- | --- | --- |
| Low | 1685.5 (10.6) | 20.3% |
| Mid | 1686.1 (12.9) | 18.3% |
| High | 1712.1 (8.4) | 16.8% |
| p-value | 0.027 | 0.003 |

F statistic p-value obtained linear regression
