# Supplementary material for: Is it possible to model the impact of calorie-reduction interventions on childhood obesity at a population level and across the range of deprivation: Evidence from the Avon Longitudinal Study of Parents and Children (ALSPAC)
Source: PLoS One. 2022 Jan 31;17(1):e0263043. doi: 10.1371/journal.pone.0263043 (PMC8803143; doi:10.1371/journal.pone.0263043)
Supplement: S2 Table — (DOCX) [file pone.0263043.s004.docx]

**S2 Table.** Relationship between baseline and time-varying confounding and exposure, mediator and outcome variables (n=10,680)

|  | **Maternal social class**  **% in lowest social class** | **Total daily calories**  **Median (SE)** | **zBMI 11 years**  **% obese** |
| --- | --- | --- | --- |
| Ethnicity |  |  |  |
| White | 29.2% | 1693.9 (5.4) | 18.7% |
| Non-white | 29.0% | 1702.5 (28.4) | 21.9% |
| p-value | 0.349 | 0.866 | 0.241 |
|  |  |  |  |
| Birthweight |  |  |  |
| Low | 30.9% | 1659.4 (12.7) | 14.9% |
| Mid | 28.9% | 1693.5 (13.5) | 18.2% |
| High | 29.0% | 1731.7 (17.8) | 26.0% |
| p-value | 0.204 | <0.001 | <0.001 |
|  |  |  |  |
| Child physical health |  |  |  |
| Sometimes/always unwell | 31.7% | 1620.4 (32.6) | 21.9% |
| Very healthy/healthy | 29.1% | 1696.4 (5.5) | 18.8% |
| p-value | 0.659 | 0.006 | 0.633 |
|  |  |  |  |
| Child activities score |  |  |  |
| Low | 30.6% | 1642.2 (16.6) | 17.9% |
| Mid | 30.2% | 1688.5 (2.7) | 18.8% |
| High | 26.0% | 1723.8 (4.2) | 19.3% |
| p-value | <0.001 | <0.001 | 0.824 |
|  |  |  |  |
| Weekly TV time |  |  |  |
| Low (<=14hrs) | 22.0% | 1698.6 (17.2) | 13.6% |
| Mid (>14hrs and <=26hrs) | 29.1% | 1697.1 (18.0) | 18.3% |
| High (>26 hours) | 33.6% | 1672.9 (22.2) | 24.9% |
| p-value | <0.001 | 0.036 | <0.001 |

F statistic p-values obtained from multinomial (maternal social class), linear (total daily calories), and logistic regressions (Obesity at 11 years).
