# Supplementary material for: Is it possible to model the impact of calorie-reduction interventions on childhood obesity at a population level and across the range of deprivation: Evidence from the Avon Longitudinal Study of Parents and Children (ALSPAC)
Source: PLoS One. 2022 Jan 31;17(1):e0263043. doi: 10.1371/journal.pone.0263043 (PMC8803143; doi:10.1371/journal.pone.0263043)
Supplement: S3 Table — (DOCX) [file pone.0263043.s005.docx]

**S3 Table.** CDE and simulation 1 by highest level of maternal education (n=10,680)

| **Scenario** | **% consuming less <=EAR (boys/girls)** | **Prevalence of obesity at 11 years (>=95^th^ centile)** | | | | **Inequalities in obesity^a^** | |
| --- | --- | --- | --- | --- | --- | --- | --- |
|  |  | **Overall**  **(% change**  **vs CDE)** | **Highest maternal education level** | | | **Risk ratio^b^**  **(CIs)** | **Risk difference^b^**  **(CIs)** |
|  |  |  | **Low**  **(% change**  **vs CDE)** | **Mid**  **(% change**  **vs CDE)** | **High**  **(% change**  **vs CDE)** |  |  |
| Control Direct Effect^c^ | | | | | | | |
|  | 44.3% / 29.3% | 18.1% | 20.7% | 18.2% | 16.1% | 1.29 (1.12 – 1.45) | 4.64 (2.30 – 6.98) |
| Simulation 1: Universal intervention to meet kcal per day recommendation (-6.1% overall), 75% | | | | | | | |
|  | 54.6% / 39.3% | 17.5% (-3.6%) | 20.0% (-3.5%) | 17.6% (-3.6%) | 15.5% (-3.7%) | 1.29 (1.13 – 1.45) | 4.51 (2.24 – 6.78) |

^a^ Relative and absolute inequalities were estimated using a continuous linear term for maternal social class.

^b^ Risk ratios and differences are likelihoods calculated with reference to non-obese group (<95^th^ centile of zBMI at age 11 years).

^c^ The effect of maternal social class on obesity prevalence at age 11 years, adjusted for baseline and time-varying confounding with mediation of total daily calories held at observed level.
