# Supplementary material for: Is it possible to model the impact of calorie-reduction interventions on childhood obesity at a population level and across the range of deprivation: Evidence from the Avon Longitudinal Study of Parents and Children (ALSPAC)
Source: PLoS One. 2022 Jan 31;17(1):e0263043. doi: 10.1371/journal.pone.0263043 (PMC8803143; doi:10.1371/journal.pone.0263043)
Supplement: S4 Table — (DOCX) [file pone.0263043.s006.docx]

**S4 Table.** CDE and simulation 1 by 100%, 75% and 30% uptake (n=10,680)

| **Scenario** | **% consuming less <=EAR**  **(boys/girls)** | **Prevalence of obesity at 11 years (>=95^th^ centile)** | | | | **Inequalities in obesity^a^** | |
| --- | --- | --- | --- | --- | --- | --- | --- |
|  |  | **Overall (% change**  **vs CDE)** | **Maternal occupational social class** | | | **Risk ratio^b^ (CIs)** | **Risk difference^b^ (CIs)** |
|  |  |  | **Low (% change**  **vs CDE)** | **Mid (% change**  **vs CDE)** | **High (% change**  **vs CDE)** |  |  |
| Control Direct Effect^c^ | | | | | | | |
|  | 44.3% / 29.3% | 18.3% | 20.3% | 18.2% | 16.7% | 1.21 (1.06 – 1.36) | 3.52 (1.15 – 5.88) |
| Simulation 1a: Universal intervention to meet kcal per day recommendation (-6.1% overall), 75% | | | | | | | |
|  | 54.6% / 39.3% | 17.7% (-3.5%) | 19.6% (-3.5%) | 17.6% (-3.5%) | 16.2% (-3.5%) | 1.21 (1.06 – 1.37) | 3.41 (1.11 – 5.71) |
| Simulation 1b: Universal intervention to meet kcal per day recommendation (-6.1%), 100% uptake | | | | | | | |
|  | 58.2% / 42.5% | 17.4% (-4.7%) | 19.3% (-4.6%) | 17.4% (-4.7%) | 15.9% (-4.8%) | 1.21 (1.06 – 1.37) | 3.39 (1.11 – 5.67) |
| Simulation 1c: Universal intervention to meet kcal per day recommendation (-6.1%), 30% uptake | | | | | | | |
|  | 48.3% / 33.3% | 18.0 (-2.1%) | 20.0% (-2.1%) | 18.0% (-2.1%) | 16.5% (-2.2%) | 1.21 (1.06 – 1.37) | 3.47 (1.13 – 5.81) |

^a^ Relative and absolute inequalities were estimated using a continuous linear term for maternal social class.

^b^ Risk ratios and differences are likelihoods calculated with reference to non-obese group (<95^th^ centile of zBMI at age 11 years).

^c^ The effect of maternal social class on obesity prevalence at age 11 years, adjusted for baseline and time-varying confounding with mediation of total daily calories held at observed level.
