# Supplementary material for: Is it possible to model the impact of calorie-reduction interventions on childhood obesity at a population level and across the range of deprivation: Evidence from the Avon Longitudinal Study of Parents and Children (ALSPAC)
Source: PLoS One. 2022 Jan 31;17(1):e0263043. doi: 10.1371/journal.pone.0263043 (PMC8803143; doi:10.1371/journal.pone.0263043)
Supplement: S5 Table — (DOCX) [file pone.0263043.s007.docx]

**S5 Table.** CDE and simulation 1 for obesity and overweight/obesity combined (n=10,680)

| **Scenario** | **% consuming less <=EAR**  **(boys/girls)** | **Prevalence of overweight and obesity at 11 years (>=85th centile)** | | | | **Inequalities in obesity^a^** | |
| --- | --- | --- | --- | --- | --- | --- | --- |
|  |  | **Overall**  **(% change**  **vs CDE)** | **Maternal occupational social class** | | | **Risk ratio^b^**  **(CIs)** | **Risk difference^b^**  **(CIs)** |
|  |  |  | **Low**  **(% change**  **vs CDE)** | **Mid**  **(% change**  **vs CDE)** | **High**  **(% change**  **vs CDE)** |  |  |
| Control Direct Effect^c^ | | | | | | | |
|  | 44.3% / 29.3% | 34.4% | 37.1% | 34.3% | 32.3% | 1.15 (1.05 – 1.24) | 4.71 (1.73 – 7.69) |
| Simulation 1: Universal intervention to meet kcal per day recommendation (-6.1% overall), 75% | | | | | | | |
|  | 54.6% / 39.3% | 33.4% | 36.0% | 33.2% | 31.4% | 1.15 (1.05 – 1.25) | 4.63 (1.69 – 7.56) |

^a^ Relative and absolute inequalities were estimated using a continuous linear term for maternal social class.

^b^ Risk ratios and differences are likelihoods calculated with reference to non-obese group (<95^th^ centile of zBMI at age 11 years).

^c^ The effect of maternal social class on obesity prevalence at age 11 years, adjusted for baseline and time-varying confounding with mediation of total daily calories held at observed level.
